# The Story of the Insane from Year to Year

**Published:** 1895-03-23

**Authors:** 


### THE STORY OF THE INSANE FROM YEAR TO YEAR.

(7th Series.)

### NORTH RIDING ASYLUM, YORK.

An increase of 17 patients has to be noted during the year, there being on December 31st 722 in the asylum. There were 184 admissions, 73 recoveries, and 80 deaths. The percentage of recoveries calculated on the admissions was 41·7, and the percentage of deaths on the average number resident was 11·1. Drink was the assigned cause of the insanity in 15 cases, and hereditary influence in 23. Religion figures as the cause in 6, love in 5, and 39 are put down as "unknown." Cerebral and spinal diseases were the causes of death in 31 and consumption in 13. During the year there was considerable pressure on the space, and a great many applications for the admission of private patients had to be refused. The connection between the North Riding and the Borough of Middlesbrough will be terminated shortly, and the removal of the borough patients will give the required accommodation. Attendant James Simpson received a pension of £38 10s. per annum after seventeen years' service. While congratulating the attendant on obtaining his pension, we are sorry to note that ill-health was the cause of his resignation. The Commissioners' report is not given. The weekly rate of maintenance is 9s. 7½d. per head.

### SALOP AND MONTGOMERY ASYLUM.

On January 1st this asylum contained 770 patients, and on December 31st there were 9 additional in residence. Of these 29 were private cases. The admissions were 191, the recoveries 62, and the deaths 78. Calculated on the admissions the recoveries work out at 32·46 per cent., and the deaths at 9·94. The recovery rate is not high, but in addition to the recoveries 28 patients were discharged relieved. Of the admissions 62 were due to hereditary influence alone or in combination, 13 to drink, and 5 to domestic trouble; 50 are stated to be of unknown origin. Dr. Strange does not agree with all the suggestions made by the Commissioners in Lunacy, but we think if he will adopt the plan recommended for the w.c.'s he will find it a great improvement, and the alteration need not necessarily entail much expense, unless those in use at present are badly constructed. We congratulate Attendant John Ashton on having obtained a pension of 15s. a week, but we feel inclined to think he ought to have had it long since, for it is questionable whether any attendant can stand the strain of asylum life for two-and-thirty years. It is no wonder he was "incapacitated by age and infirmity." The visitors say that "the devotion to duty on the part of the superintendent deserves special praise." The weekly rate is 7s. 9d. per head for in-county patients. Dr. Strange must be a good farmer, for he made £776 profit last year.

